# Trends in prevalence and factors associated with unintended pregnancies in Zambia (2001–2018)

**DOI:** 10.1186/s12884-024-06311-7

**Published:** 2024-02-21

**Authors:** Milika Sikaluzwe, Million Phiri, Musonda Lemba, Liness Shasha, Mikidadi Muhanga

**Affiliations:** 1 Department of Demography, Population Sciences, Monitoring and Evaluation, School of Humanities and Social Sciences, University of Zambia, Lusaka, Zambia; 2 Demography and Population Studies Programme, Schools of Public Health and Social Sciences, University of the Witwatersrand, Johannesburg, South Africa; 3 Department of the Development and Strategic Studies, College of Social Sciences and Humanities, Sokoine University of Agriculture, Morogoro, Tanzania

**Keywords:** Women, Reproductive health, Unintended pregnancy, Family planning, Zambia

## Abstract

**Background:**

Unintended pregnancies can pose significant public health concerns for both maternal and child health because of their associated risks and implications. Experience of unintended pregnancies may lead to delay in seeking antenatal care, thus leading to increased risk of complications during pregnancy and childbirth. Globally, the prevalence of unintended pregnancies has declined. However, the problem remains acute in sub-Saharan Africa. This study was conducted to examine the factors associated with an experience of unintended pregnancy among women of reproductive ages in Zambia.

**Methods:**

This study used secondary data from the Zambia Demographic and Health Surveys (ZDHSs) which were conducted between 2001 and 2018. A pooled weighted sample of 4,090 pregnant women of reproductive age 15–49 years at the time of the survey was included in the analysis. Multivariable binary logistic regression model was employed to examine the association between independent correlates and experience of unintended pregnancy. All statistical analyses were conducted using Stata software.

**Results:**

Findings show that the proportion of women of reproductive age who experienced unintended pregnancy in Zambia declined from 50.4% (95% CI: 47.1, 53.8) in 2001 to 45.2% (95% CI: 40.5, 49.9) in 2018. The decline in the prevalence of unintended pregnancy is more pronounced among women age groups 25–29 years and 30–34 years. Increasing age was associated with an increased risk of experiencing unintended pregnancies. On the other hand, women who were living in rural areas (aOR = 0.76; 95% CI: 0.58, 1.00) and those with tertiary education (aOR = 0.46; 95% CI: 0.26, 0.80) were less likely to experience an unintended pregnancy. Women who desired a large family (aOR = 0.45; 95% CI: 0.24, 0.85) and those who watched television (aOR = 0.75; 95% CI: 0.59, 0.94) had lower odds of experiencing unintended pregnancies.

**Conclusions:**

The study has established that the prevalence of unintended pregnancy is still high in Zambia. Women’s age, place of residence, level of education, desired family size and exposure to media were associated with the risk of experiencing an unintended pregnancy. Enhancing access to family planning services and commodities targeting women with low education levels will be key to further reduce unintended pregnancies.

**Supplementary Information:**

The online version contains supplementary material available at 10.1186/s12884-024-06311-7.

## Introduction

Unintended pregnancy is defined as pregnancy that either happens earlier or later than occurred (mistimed) or not needed at all (unintended) [1]. The occurrence of unintended pregnancies has for a long time been of public health concern among demographers and public health practitioners due to its direct effect on women’s health [2–4]. Unintended pregnancy is still a social and health concern despite global improvements in family planning (FP) service provision and utilisation [5–7]. Globally, 38% of pregnancies are unintended [8]. Although the prevalence of unintended pregnancies has declined globally [9], in sub-Saharan Africa (SSA), unintended pregnancy remains high and accounts for more than a quarter of the 40 million pregnancies that occur annually [8]. Unintended pregnancies can have implications at the individual and community levels, these include socioeconomic, psychological, and maternal health complications [4, 10, 11]. Globally, there has been a decline in the prevalence of unintended pregnancy from 51% in 2008 to 45% in 2011 [9, 12]. 49% of women in the reproductive age range were using contraception in 2019 at the global level, an increase from 42% in 1990 [13]. One of the best ways to lower the chance of unintended pregnancies is to use contraceptive methods, which allow women and couples to schedule when and how many children they want to have [14]. The decline in the prevalence of unintended pregnancy is partly attributed to the increase in access to FP commodities and services, which has led to improved uptake of contraceptives among women. The observed global decline in the prevalence of unintended pregnancies is an important trend with significant implications for public health, sexual and reproductive health and rights (SRHR), gender equality, and family well-being [9, 12]. Despite this general decline and the widespread availability of various FP methods, the phenomenon is reported to remain high in SSA [3, 8, 15–18].

Contraceptive use among women of reproductive age has increased from 29.9% in 2007 to 35.4% in 2018 in Zambia, while the prevalence of unplanned births and pregnancies is still high at 38% in 2018 [19]. Unintended pregnancy predisposes women to several risks, such as unsafe abortion, maternal death, malnutrition, mental illness, and vertical transmission of HIV to children [20–22]. Furthermore, research show that the risk of unintended pregnancy in SSA continues to be high and unsafe, which predisposes approximately 1 in 16 women to psychosocial effects of morbidity and mortality [23, 24]. Additionally, it increases stress levels, impacts negatively on women’s quality of life, and threatens the economic status of families [25, 26].

Studies that have been conducted in SSA countries show heterogeneity in demographic and socio-economic factors that are associated with the experience of unintended pregnancy [1, 8, 17, 27–29]. These factors operate at individual, household, and community levels. A study conducted in Ethiopia in 2013 found that ever use of contraception, having five or more children, and two or more births in the past five years was associated with unintended pregnancy [2]. A similar study conducted in 2019 in Ghana found that women’s age, level of education, and household wealth status were major determinants of unintended pregnancy [16]. Women’s age, place of residence, and employment status were found to be associated with the experience of unintended pregnancies in Kenya [8].

To improve maternal and child health outcomes in Zambia, the Ministry of Health (MoH), in collaboration with partners including the United States for International Development (USAID), the United Nations Population Fund (UNFPA) and the European Union-funded Millennium Development Goal Initiative (MDGi) have developed various health policy strategies and interventions aimed at improving access and utilization of FP services. Notable strategies include: the Zambia FP Costed Implementation Plan and Business Case 2021–2026 [30], the National Health Strategic Plan 2017–2021, the Reproductive, Maternal, New-born, Child and Adolescent Health and Nutrition Communication and Advocacy Strategy 2018–2021 [31] and the National Adolescent Health Strategic Plan [32].

The review of the literature shows that there is little evidence of research on factors that are associated with unintended pregnancies among women of reproductive age in Zambia. Empirical information on the determinants of unintended pregnancies in Zambia would contribute to the ongoing national efforts by demographers and public health practitioners to effectively address the effects of this public health concern. This study was therefore conducted to examine the factors associated with the experience of unintended pregnancies among women of reproductive age in Zambia. Findings from this study will inform designing of strategies and interventions to address the problem of unintended pregnancies in Zambia.

## Methods and data

### Data source

**Fig. 1:**
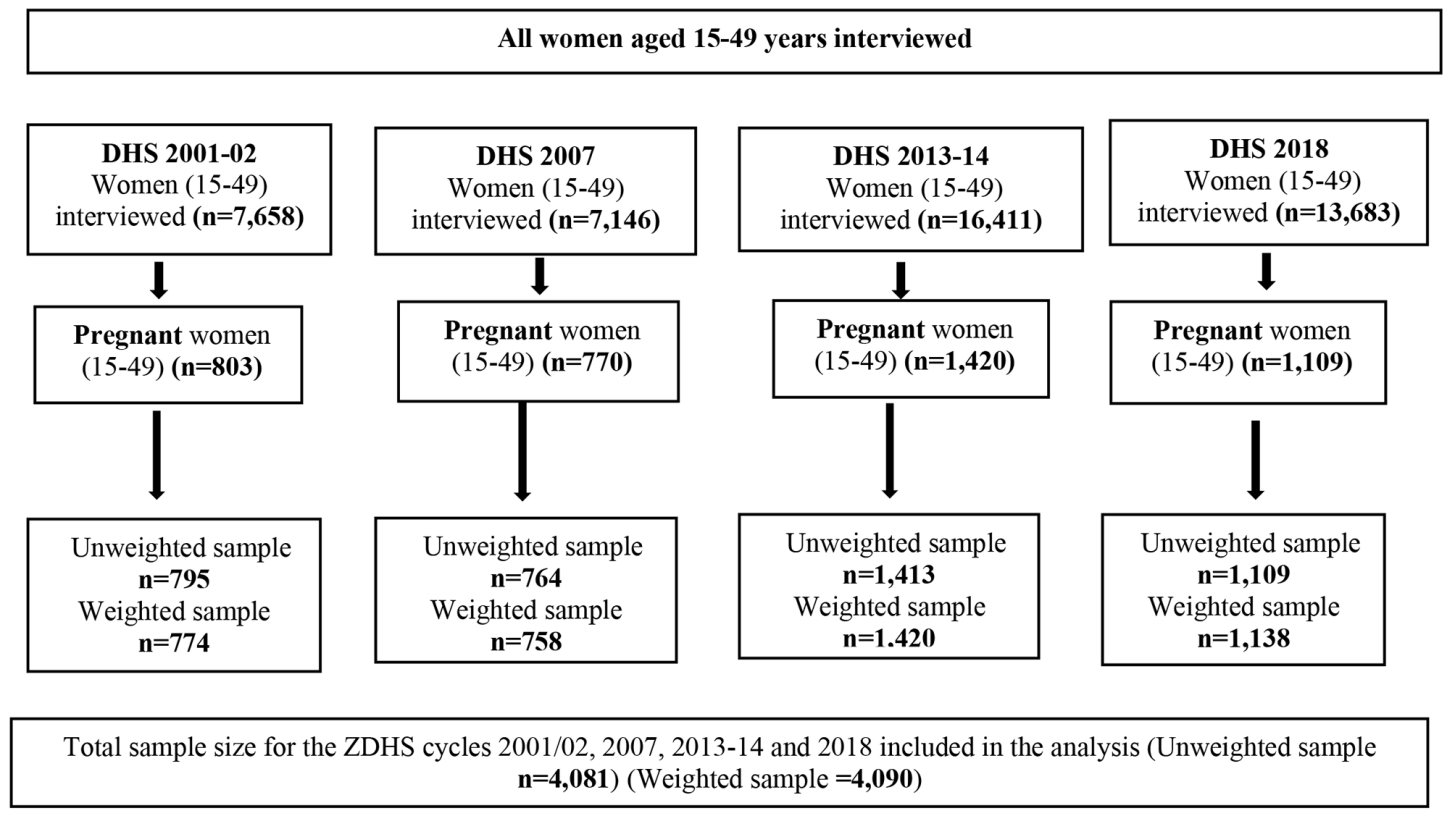
Sample derivation and inclusion criteria

Secondary data from the Zambia Demographic and Health Survey (ZDHS) conducted between 2001 and 2018 was used in this study. Specifically, the study used the Individual Recode (IR) files, which contains the responses of women aged 15–49. The Demographic and Health Survey (DHS) is a nationwide survey that is carried out across low-and middle-income countries (LMICs) every five-years [33] and collects data on several indicators, unintended pregnancy inclusive. The DHS has been an essential source of data on issues, surrounding sexual and reproductive health in LMICs as it gathers data on several reproductive health indicators such as marriage, sexual activity, fertility, fertility preferences, and FP [33]. A stratified two-stage sampling approach was employed in selecting the sample for each survey. The DHS collected data using four standard questionnaires, namely; Woman, Man, Household and Biomarker. The data used in this study was collected using the Woman questionnaire. The Woman questionnaire was administered to all women aged 15–49 who were found in all selected households. A pooled weighted sample of 4,090 women aged 15–49 years, who were pregnant during the survey and had complete cases on all the variables of interest, were included in the analysis. The distribution of sample sizes per survey year is provided in Fig. 1.

### Measurement of variables

#### Outcome variable

The outcome variable for this study was unintended pregnancy. Unintended pregnancy is defined as “pregnancy that happens earlier or later than occurred (mistimed) or not needed at all (unintended)” [1]. The DHS variable that was used to collect this information was classified with three response categories; (i) ‘pregnancy wanted then’, (ii) pregnancy wanted later and (iii) pregnancy not wanted at all. We created a binary variable from the initial variable with the classification “0” representing pregnancy wanted then and “1” representing pregnancy wanted later or not wanted at all.

### Independent variables

Based on existing literature, 11 explanatory variables were selected at individual and household level [1, 34, 35]. The variables were selected based on their importance in explaining the experience of unintended pregnancy, as reported by prior studies conducted in SSA counties. These include age of a woman, residence, education level, household wealth index, employment status, age at first sex, desired family size, exposure to mass-media FP messages, frequency of listening to the radio, frequency of watching television (TV), and frequency of reading newspaper.

Age was categorized as 15–24 years, 25–29 years, 30–34 years, and 35–49 years; age at first sex (below 15 years, 15–24 years, and 25 years+); desired family size (0–1 child, 2–3 children, 4 + children). Other variables included residence (urban, rural); education level (no education, primary, secondary, and tertiary); household wealth index (poor, medium, rich); employment status (employed, unemployed); exposure to mass-media FP messages (no, yes); frequency of listening to the radio (no, yes); frequency of watching TV (no, yes); frequency of reading newspaper (no, yes).

### Statistical analysis

Data analysis was done in three stages; descriptive, bivariate, and multivariable. All analyses were performed using Stata version 17 software, with a 5% level of significance. At a descriptive stage, frequencies and percent distributions of selected background characteristics of women were presented. Trend analysis of unintended pregnancies was conducted relative to the respective survey years, between 2001 and 2018. At the bivariate level, cross-tabulations with chi-square tests were used to analyse the association between unintended pregnancy and the selected independent variables. To assess the effects of several individual-level factors on unintended pregnancies in Zambia, a multivariable binary logistic regression model was applied on pooled data for all the four surveys. Adjusted odds ratios (AOR) with corresponding 95% confidence intervals (CI) were reported. All covariates from the bivariate analyses were included in the multivariable logistic regression analysis, regardless of their significant levels. Multicollinearity was assessed on all independent variables using the “estat vif” command in Stata. Results show no concerns of multicollinearity (Supplementary Table 1).

### Ethical approval

The data analysed in this study is available in the public domain at https://dhsprogram.com/. Permission to the data was obtained from the DHS program. All data used did not contain any identifying information. The original Zambia DHS 2001–2018 Biomarker and survey protocols were approved by Tropical Disease and Research Center (TDRC) and the Research Ethics Review Board of the Centers for Disease Control and Prevention (CDC) Atlanta. Thus, all data collection methods were carried out in accordance with relevant ethical guidelines and regulations. The DHS protocols ensured that all participants older than 18 years who were enrolled in the DHS gave their informed consent during enumeration. Additionally, parents or guardians of all participants aged 15 to 17 gave informed consent before the legal minors were asked for their assent.

## Results

### Characteristics of the study sample

A total of 4,081 women were included in the analysis across the four ZDHSs. Nearly 35% of the women came from the 2013 ZDHS, while 27.8% came from the 2018 survey. Nearly 19% came from the 2001 survey and 18.5% came from the 2007 survey. Table 1 shows the distribution of women included in the analysis by background characteristics across all the survey years. The distribution of respondents across ages shows that most of them (below 50%) were aged between 15 and 24 years in all the four survey years. In terms of residence, the distribution showed that in all the survey years, most of the respondents were from rural areas (70.8%, 69.4%, 61.7% and 63.9% respectively). Regarding the highest level of education, the majority of respondents had attained only primary school level education across all the survey years, 2001, 2007, 2013 and 2018 (64.0%, 63.2%, 50.3% and 48.5% respectively). The results in Table 1 further show that most of the respondents across all the survey years, 2001 (43.8%), 2007 (42.5%), 2013 (43.0%) and 2018 (44.5%) came from poor households. In terms of employment status, 58.1% in 2007, 52.7% in 2013 and 59.9% in 2018 were not working. Table 1 further shows that in all survey years 2001 (57.8%), 2007 (56.4%), 2013 (55.8%) and 2018 (82.5%), most of the women had their first sexual debut in the age group 15–24. Majority of the women, ranging from 53.7 to 76.7%, had no exposure to FP messages in all the survey years.

**Table 1:** Percent distribution of background characteristics of currently pregnant women (15–49 years), 2001–2018 DHS, Zambia

Background Characteristics	DHS 2001 (*N* = 774)	DHS 2007 (*N* = 758)	DHS 2013 (*N* = 1,420)	DHS 2018 (*N* = 1,138)
	N (%)	N (%)	N (%)	N (%)
**Age**				
15–24	365 (47.1)	326 (43.0)	583 (41.1)	540 (47.5)
25–29	211 (27.3)	179 (23.7)	326 (23.0)	252 (22.1)
30–34	112 (14.4)	148 (19.5)	287 (20.2)	168 (14.7)
35–49	86 (11.1)	104 (13.8)	224 (15.8)	178 (15.7)
**Residence**				
Urban	226 (29.2)	232 (30.6)	544 (38.3)	411 (36.1)
Rural	548 (70.8)	526 (69.4)	875 (61.7)	727 (63.9)
**Education level**				
No education	101 (13.0)	79 (10.4)	163 (11.5)	98 (8.6)
Primary	495 (64.0)	479 (63.2)	713 (50.3)	552 (48.5)
Secondary	166 (21.5)	173 (22.8)	487 (34.3)	429 (37.6)
Tertiary	11 (1.5)	27 (3.5)	56 (3.9)	60 (5.3)
**Household wealth index**				
Poor	339 (43.8)	322 (42.5)	611 (43.0)	507 (44.5)
Medium	182 (23.5)	173 (22.9)	266 (18.7)	219 (19.3)
Rich	253 (32.7)	263 (34.7)	543 (38.3)	412 (36.2)
**Employments status**				
Not working	309 (40.0)	439 (58.1)	745 (52.7)	681 (59.9)
Working	464 (60.0)	317 (41.9)	668 (47.3)	457 (40.1)
**Age at first sex**				
Below 15	107 (13.8)	76 (10.0)	134 (9.4)	189 (16.6)
15–24	448 (57.8)	428 (56.4)	792 (55.8)	939 (82.5)
25+	219 (28.3)	254 (33.6)	494 (34.8)	10 (0.9)
**Exposure to mass-media FP Messages**				
No	416 (53.7)	437 (57.7)	955 (67.2)	873 (76.7)
Yes	358 (46.3)	321 (42.3)	465 (32.8)	266 (23.3)

### Distribution of unintended pregnancies according to background characteristics

Table 2 shows the trends in the prevalence of unintended pregnancies according to different socio-economic and demographic variables. The overall trend in the prevalence of unintended pregnancy shows a reduction from 50.4% in 2001 to 45.2% in 2018. 

Residence, education level, household wealth index, age at first sex and frequency of reading newspapers were found to be statistically associated with unintended pregnancies (*p* < 0.05) in 2001, 2007, and 2018 DHS. All the trends show a decline in unintended pregnancies in both rural and urban areas; women living in urban areas had higher rates of unintended pregnancies compared with their rural counterparts (57.7% versus 47.4%) in 2001.

Regarding the level of education, unintended pregnancies were highest among women with a secondary level of education. The prevalence of pregnancies reduced significantly among women with tertiary level of education from 39.9% in 2001 to 22.1% in 2018. In terms of household wealth status, in 2013 and 2018 women from households classified as poor or middle had higher rates of unintended pregnancies compared to those from the households classified as rich. Trends show a decrease in unintended pregnancies from 59.2% in 2001 to 40.4% in 2018 among women coming from rich households.

In terms of age at first sex, unintended pregnancies reduced across survey years. The prevalence was higher among women who had their first sex before age 15. Furthermore, unintended pregnancies increased among women who read a newspaper from 47.3% in 2001 to 51.1% in 2018 (Table 2). However, the prevalence declined among women who had exposure to watching television (54.3% in 2001 to 41.2% in 2018).

**Table 2:** Percent distribution of unintended pregnancies by background characteristics, 2001–2018 DHS, Zambia

	Number of unintended pregnancies			
Background Characteristics	DHS 2001 (*N* = 774)	DHS 2007 (*N* = 758)	DHS 2013 (*N* = 1,420)	DHS 2018 (*N* = 1,138)
	N (%)	N (%)	N (%)	N (%)
**Age**	**	*	***	*
15–24	365 (44.2)	326 (46.8)	583 (46.1)	540 (44.4)
25–29	211 (52.0)	179 (52.1)	326 (37.1)	252 (38.5)
30–34	112 (54.5)	148 (53.6)	287 (38.2)	168 (42.4)
35–49	86 (67.7)	104 (65.2)	224 (61.6)	178 (59.5)
**Residence**	*	**ns**	**ns**	**ns**
Urban	226 (57.7)	232 (57.9)	544 (43.9)	411 (46.4)
Rural	548 (47.4)	526 (49.3)	875 (45.5)	727 (44.5)
**Education level**	**ns**	*	**ns**	*
No education	101 (45.0)	79 (45.8)	163 (44.2)	98 (43.7)
Primary	495 (52.5)	479 (49.5)	713 (45.4)	552 (46.1)
Secondary	166 (48.4)	173 (63.0)	487 (46.0)	429 (47.6)
Tertiary	11 (39.9)	27 (41.8)	56 (29.7)	60 (22.1)
**Household Wealth Index**	**	**ns**	**ns**	**
Poor	339 (46.6)	322 (48.2)	611 (46.0)	507 (43.4)
Medium	182 (45.5)	173 (50.6)	266 (48.8)	219 (58.2)
Rich	253 (59.2)	263 (57.3)	543 (41.7)	412 (40.4)
**Employment Status**	**ns**	**ns**	**ns**	**ns**
Not working	309 (52.9)	439 (51.6)	745 (44.3)	681 (44.8)
Working	464 (48.8)	317 (52.6)	668 (45.5)	457 (45.8)
**Age at first sex**	*	**ns**	**ns**	**ns**
Below 15	107 (53.4)	76 (57.0)	134 (45.8)	189 (48.2)
15–24	448 (53.8)	428 (52.7)	792 (45.1)	939 (44.8)
25+	219 (42.1)	254 (49.0)	494 (44.3)	10 (23.7)
**Desired family size**	**ns**	**ns**	**ns**	**ns**
0–1	8 (82.9)	13 (81.5)	24 (43.6)	10 (68.0)
2–3	151 (53.4)	157 (56.7)	299 (42.7)	237 (49.9)
4+	614 (49.3)	588 (50.0)	1,097 (45.5)	892 (43.7)
**Exposure to mass-media FP Messages**	**ns**	**ns**	**ns**	**ns**
No	416 (48.5)	437 (49.7)	955 (43.9)	873 (46.9)
Yes	358 (52.7)	321 (55.0)	465 (47.0)	266 (39.5)
**Exposure to listening to radio**	**Ns**	**Ns**	**ns**	**Ns**
No	361 (47.5)	258 (47.2)	600 (42.1)	626 (46.5)
Yes	412 (53.1)	500 (54.3)	820 (46.9)	513 (43.6)
**Exposure of watching television**	**Ns**	**Ns**	**ns**	**Ns**
No	589 (49.2)	560 (51.2)	878 (46.2)	746 (47.3)
Yes	185 (54.3)	198 (53.8)	541 (42.7)	392 (41.2)
**Exposure of reading newspaper**	**Ns**	***	**ns**	**Ns**
No	654 (51.0)	554 (48.0)	1,037 (45.7)	963 (44.1)
Yes	118 (47.3)	202 (63.2)	380 (42.5)	176 (51.1)
**Total**	**774 (50.4)**	**758 (51.9)**	**1,420 (44.9)**	**1,138 (45.2)**

*** *p* < 0.001; ** = *p* < 0.01; * = *p* < 0.05; ns = non-significant

### Determinants of unintended pregnancies

After considering all covariates in the multivariable model, results show that the age of a woman, residence, education, desired family size and frequency of watching TV were significantly associated with an experience of unintended pregnancies among women of reproductive age in Zambia (Table 3).

Study results show that increasing age is associated with an increased risk of unintended pregnancies. Women in age groups 15–24 (aOR = 0.44; 95% CI: 0.35, 0.55), 25–29 (aOR = 0.44; 95% CI: 0.35, 0.57), 30–34 (aOR = 0.48; 95% CI: 0.38, 0.62), respectively were less likely to experience unintended pregnancies compared to those in the age group 35–49. The study findings also revealed that women who were residing in rural areas (aOR = 0.76; 95% CI: 0.58, 1.00) were 24% less likely to experience unintended pregnancies compared to their counterparts residing in urban areas. Women with tertiary education (aOR = 0.46; 95% CI: 0.26, 0.80) had lower odds of experiencing unintended pregnancies compared with women with no level of education. Results also show that women who desired a large family (4 or more children) were 55% less likely to experience unintended pregnancies compared to those who desired small family size. We also observed that women who watched television had lower odds (aOR = 0.75; 95% CI: 0.59, 0.94) of experiencing unintended pregnancies compared with those that had no exposure (Table 3).

**Table 3:** Multivariable logistic regression analysis examining variations of unintended pregnancies among women 15–49 in Zambia, DHS 2001–2018

Background Characteristics	Unintended pregnancies	
	Adjusted odds ratio	[95% CI]
	(aOR)	
**Age**		
15–24	0.44***	(0.35 0.55)
25–29	0.44***	(0.35 0.57)
30–34	0.48***	(0.38 0.62)
35–49	**Ref**	
**Residence**		
Urban	**Ref**	
Rural	0.76*	(0.58 1.00)
**Education level**		
No education	**Ref**	
Primary	1.12	(0.88 1.43)
Secondary	1.15	(0.87 1.52)
Tertiary	0.46**	(0.26 0.80)
**Household Wealth index**		
Poor	**Ref**	
Medium	1.12	(0.92 1.39)
Rich	0.95	(0.74 1.21)
**Employment status**		
Not working	**Ref**	
Working	1.02	(0.87 1.19)
**Age at first sex**		
Below 15	**Ref**	
15–24	0.90	(0.72 1.14)
25+	0.77	(0.59 1.01)
**Desired family size**		
0–1	**Ref**	
2–3	0.57	(0.30 1.10)
4+	0.45*	(0.24 0.85)
**Exposure to mass-media FP messages**		
No	**Ref**	
Yes	1.08	(0.90 1.29)
**Frequency of listening to a radio**		
No	**Ref**	
Yes	1.17	(0.98 1.39)
**Frequency of watching television**		
No	**Ref**	
Yes	0.75*	(0.59 0.94)
**Frequency of reading newspaper**		
No	**Ref**	
Yes	1.16	(0.94 1.42)

*** *p* < 0.001; ** = *p* < 0.01; * = *p* < 0.05;

## Discussion

This study analysed the determinants of unintended pregnancies among women of reproductive age in Zambia. The study applied a multivariable binary logistic regression model on pooled Zambia Demographic and Health Survey data collected between 2001 and 2018. This study shows disparities in the experience of unintended pregnancies among different sociodemographic characteristics. A comprehensive understanding of factors that influence experience of unintended pregnancies has significant implications for designing interventions to improve access and utilisation of FP services among women in Zambia.

Our study reveals that the proportion of women who experienced unintended pregnancies in Zambia has declined from 50.4% in 2001 to 45.2% in 2018. The prevalence of unintended pregnancies found in this analysis is similar to what was previously found in similar studies conducted in other countries in SSA. Palamuleni and Adebowale (2014) reported that nearly 43% of the pregnancies were unintended in Malawi [17], Ameyaw et al., (2019) found that 54.5% of pregnancies in Namibia were unplanned [16] and 46.6% of pregnancies in the Gambia were unintended or mistimed in 2020 [18]. The declining trend in the prevalence of unintended pregnancies in Zambia could be attributed to the increase in the proportion of women using modern contraceptive methods which has resulted in a reduction in the prevalence of unmet need for family planning [19, 36–38].

Our study found that the older age of a woman was associated with increasing odds of unintended pregnancies. This finding shows a positive association between the age of a woman and the risk of unintended pregnancy. A finding of this study resonates with previous studies conducted in other settings [16, 17, 27]. This could be attributed to the low contraceptive use among older women in Zambia when compared to young women [37, 39]. Thus, they were more likely to have unintended pregnancies. Surprisingly, our study found that women who were residing in rural areas were less likely to have unintended pregnancies compared to their counterparts living in urban areas. These findings contradict what was reported by a study conducted in Uganda [3]. One reason for our observed finding is that women living in rural areas are more likely to prefer a large family size as such, less likely to report a pregnancy as unintended. Literature shows that women in rural areas prefer large family sizes because they see children as a source of labour and wealth [40, 41].

The existing evidence in the literature makes clear how education level affects the experience of unintended pregnancies [42–44]. In this analysis, there were differences in proportions of women experiencing unintended pregnancies by education level such that women with tertiary education experienced a lower prevalence of unintended pregnancies. The most plausible explanation is that highly educated women are more empowered to manage their sexual and reproductive health (SRH) issues than their less educated counterparts. This suggests that increasing education level could impact access and utilization of contraception among women of reproductive age in Zambia. The finding is supported by an earlier study conducted in Ghana [27] which reported education as a predictor of unintended pregnancy. This finding implies that promoting women’s access to education is key to improve maternal and child health.

The study also revealed that women who frequently watched television had a reduced risk of experiencing unintended pregnancies. Many studies have documented the power of mass media as an effective tool for disseminating SRHR information and FP information [45–48]. Increased exposure to media is expected to contribute to women having more knowledge about access and benefits of using FP services. Using social media has been associated with easy access to SRH information, which can help young women to know how to plan a pregnancy or know where to access FP services [49, 50]. This is more pronounced in urban settings where access to internet is relatively easy compared to rural settings [51, 52]. Hence, access to social media can help reduce unintended pregnancy rates because of enhanced access to SRH information that helps women to make informed reproductive health decisions. This finding, therefore, underscore the need to strengthen the dissemination of FP messaging via mass media, especially among marginalized communities such as rural areas. However, practitioners must ensure that online safety and child safeguarding is paramount in the delivery of SRHR services through online or social media platforms. As these platforms increasingly serve as channels for disseminating SRHR information and support, it is crucial to create secure and protective digital environments.

Social norms influence SRH of all community members, including women. Social norms shape cultural expectations and societal perceptions surrounding fertility and FP [53, 54]. These norms may prescribe idealized timelines for childbearing, often influencing women to align their reproductive choices with prevailing community standards. The desire to conform to established norms regarding family size and timing of childbearing can negatively impact decision making on contraception use, hence leading to unintended pregnancy [55, 56].

Another strategy that would be key in preventing the experience of unintended pregnancies is implementing age-appropriate comprehensive sexuality education in schools. By offering a curriculum that addresses various aspects of SRH, including contraception, consent, and communication skills, students are equipped with the knowledge and skills necessary to make responsible and informed choices about their bodies and relationships [57–60]. Age-appropriate, comprehensive sexuality education is a school-based approach that has resulted in positive SRHR outcomes for adolescents [61, 62].

Furthermore, ensuring access to safe abortion care is a crucial strategy for addressing unintended pregnancies and promoting SRHR. When individuals face unintended pregnancies, having access to safe and legal abortion services provides them with the opportunity to make informed decisions about their reproductive futures. Access to safe abortion care reduces women’s risk of unsafe abortion, which in turn, reduces the associated risks of maternal mortality and other health complications [63, 64]. Moreover, access to safe abortion care empowers women to exercise control over their reproductive choices.

The design and implementation of FP programming in Zambia should take into account the context where contraceptive choice and pregnancy planning information is provided to young women. For example, since social media has been found to significantly affect unintended pregnancy rates, especially for urban women and younger women who have increasingly higher access to smart phones, social media, and the internet. Socialisation agents, such as schools, could be important facilitators for health issues, sexual health knowledge (SHK) inclusive. Kaale & Muhanga (2017) in their study in Tanzania have showed how low SHK has resulted into unintended pregnancy among secondary school students [65, 66]. The FP programming in Zambia can significantly channel its intended interventions through schools due to their potential as socialisation agents. The need is obvious in terms of the FP programming to be multi-sectoral by involving innumerable stakeholders such as policymakers, government officials, donors, NGOs, women-led organizations, and religious leaders.

Although the study has provided useful findings to inform factors associated with an experience of unintended pregnancies in Zambia, further research may be needed to inform designing of interventions to effectively address the problem. An inclusion of community level factors in the analysis as well as conducting a qualitative analysis could be key to have an in-depth understanding of factors that should inform designing of health interventions to resolve the problem.

### Strengths and limitations

Because the study comprised a nationally representative sample of Zambian women of childbearing age, the current analysis results can apply to the entire population of women in the reproductive age group in Zambia. The use of the DHS dataset allowed for exploration of women’s reproductive health behavior that is useful to inform the strengthening of FP programming in Zambia. A wide range of factors was assessed in this study to strengthen the associations observed. However, because of the cross-sectional nature of the data, causality cannot be inferred from this study.

## Conclusion

The study has established that, even though the prevalence of unintended pregnancies is declining in Zambia; it is still high. Women’s age, residence, level of education, desired family size and exposure to watching television were found to be significantly associated with the experience of unintended pregnancy among women of reproductive age in Zambia. Therefore, there is a need to enhance access and utilization of FP services, especially among adolescents and young women, in order to reduce further the prevalence of unintended pregnancies in Zambia. Furthermore, practitioners’/policy makers should promote and expand access to safe abortion care for women experiencing unintended pregnancies as a crucial aspect of health care, ensuring the health, well-being, and rights of all people experiencing unintended pregnancies.

### Electronic supplementary material

Below is the link to the electronic supplementary material.

Supplementary Material 1

## Data Availability

Data used in our study is publicly available at the IPUMS DHS or DHS program websites https://www.idhsdata.org/idhs/, https://dhsprogram.com/.

## Abbreviations

CI: Confidence Interval
EA: Enumeration Area
FP: Family Planning
SDG: Sustainable Development Goal
SRHR: Sexual and Reproductive Health Rights
SSA: Sub-Saharan Africa
UNFPA: United Nations Population Fund
UNICEF: United Nations Children Fund
USAID: United States Aid for International Development
WHO: World Health Organisation
ZDHS: Zambia Demographic and Health Survey
