# Supplementary material for: Trends in prevalence and factors associated with unintended pregnancies in Zambia (2001–2018)
Source: BMC Pregnancy Childbirth. 2024 Feb 21;24:148. doi: 10.1186/s12884-024-06311-7 (PMC10880343; doi:10.1186/s12884-024-06311-7)
Supplement: Supplementary file 1 — Supplementary Material 1 [file 12884_2024_6311_MOESM1_ESM.docx]

| **Supplementary Table 1: Multicollinearity test** |  |
| --- | --- |
| **Variable** | **VIF** |
| Age | 1.02 |
| Residence | 1.91 |
| Education level | 1.60 |
| Household wealth status | 2.41 |
| Employment status | 1.03 |
| Age at first sex | 1.16 |
| Desired family size | 1.10 |
| Exposure to mass-media FP messages | 1.37 |
| Frequency of listening to radio | 1.35 |
| Frequency of watching TV | 1.81 |
| Frequency of reading newspaper | 1.36 |
| **VIF; variance inflation factor** |  |
